# Genome-wide characterization and analysis of Golden 2-Like transcription factors related to leaf chlorophyll synthesis in diploid and triploid *Eucalyptus urophylla*

**DOI:** 10.3389/fpls.2022.952877

**Published:** 2022-07-28

**Authors:** Zhao Liu, Tao Xiong, Yingwei Zhao, Bingfa Qiu, Hao Chen, Xiangyang Kang, Jun Yang

**Affiliations:** ^1^National Engineering Research Center of Tree Breeding and Ecological Restoration, College of Biological Sciences and Technology, Beijing Forestry University, Beijing, China; ^2^Key Laboratory of Genetics and Breeding in Forest Trees and Ornamental Plants, Ministry of Education, Beijing Forestry University, Beijing, China; ^3^Beijing Laboratory of Urban and Rural Ecological Environment, Beijing Forestry University, Beijing, China; ^4^Guangxi Dongmen Forest Farm, Chongzuo, China

**Keywords:** chlorophyll synthesis, co-expression networks, *EgrGLK*, miRNA, polyploid, transcriptome analysis

## Abstract

Golden 2-Like (*GLK*) transcription factors play a crucial role in chloroplast development and chlorophyll synthesis in many plant taxa. To date, no systematic analysis of *GLK* transcription factors in tree species has been conducted. In this study, 40 *EgrGLK* genes in the *Eucalyptus grandis* genome were identified and divided into seven groups based on the gene structure and motif composition. The *EgrGLK* genes were mapped to 11 chromosomes and the distribution of genes on chromosome was uneven. Phylogenetic analysis of *GLK* proteins between *E. grandis* and other species provided information for the high evolutionary conservation of *GLK* genes among different species. Prediction of *cis*-regulatory elements indicated that the *EgrGLK* genes were involved in development, light response, and hormone response. Based on the finding that the content of chlorophyll in mature leaves was the highest, and leaf chlorophyll content of triploid *Eucalyptus urophylla* was higher than that of the diploid control, *EgrGLK* expression pattern in leaves of triploid and diploid *E. urophylla* was examined by means of transcriptome analysis. Differential expression of *EgrGLK* genes in leaves of *E. urophylla* of different ploidies was consistent with the trend in chlorophyll content. To further explore the relationship between *EgrGLK* expression and chlorophyll synthesis, co-expression networks were generated, which indicated that *EgrGLK* genes may have a positive regulatory relationship with chlorophyll synthesis. In addition, three *EgrGLK* genes that may play an important role in chlorophyll synthesis were identified in the co-expression networks. And the prediction of miRNAs targeting *EgrGLK* genes showed that miRNAs might play an important role in the regulation of *EgrGLK* gene expression. This research provides valuable information for further functional characterization of *GLK* genes in *Eucalyptus*.

## Introduction

Golden 2-Like (*GLK*) transcription factors are members of the GARP family (Riechmann et al., 2000; Xiao et al., 2019). Most *GLK* genes contain two highly conserved domains: a MYB DNA-binding domain and a C-terminal (GCT) box (Rossini et al., 2001). A *GLK* gene was first identified in maize (*Zea mays* L.) and, subsequently, numerous *GLK* genes were detected in *Arabidopsis thaliana*, rice (*Oryza sativa* L.), and tomato (*Solanum lycopersicum*; Fitter et al., 2002; Powell et al., 2012; Bhutia et al., 2020). The *GLK* transcription factors play crucial roles in chloroplast development and chlorophyll synthesis in many plant taxa (Bravo-Garcia et al., 2009; Chen et al., 2016).

Although the function of *GLK* genes is conserved, different genetic regulatory mechanisms may operate in different species. There are three types of chloroplasts in C_4_ plants: C_4_ bundle sheath cells, C_4_ and C_3_ mesophyll cells (Langdale and Nelson, 1991). The spatially tissue-specific expression of different *GLK* genes in maize might represent a specialization required for the development of distinct bundle sheath and mesophyll chloroplasts (Rossini et al., 2001). However, in *Cleome gynandra*, different *GLK* genes are both expressed in bundle sheath and mesophyll cells (Wang et al., 2013). In the C_3_ plant *Arabidopsis*, *GLK* genes play a redundant role in regulating chloroplast development. Single-insertion mutants showed normal phenotypes in most photosynthetic tissues, whereas in double mutants all photosynthetic tissues and chloroplasts were pale green (Fitter et al., 2002). In addition, two typical *GLK* genes were found in tomato, both of which are expressed in the leaves, but only one is predominantly expressed in fruit (Powell et al., 2012; Nguyen et al., 2014). In many plant species, *GLK* genes act as transcriptional regulators of chloroplast development. However, to date, no study of *GLK* genes in forest tree species has been conducted.

*Eucalyptus* is a genus of fast-growing tree species that are widely planted around the world (Booth et al., 2017; Deng et al., 2020). These trees provide raw materials for pulp and paper manufacturing, and have the advantage of fixing large amounts of atmospheric carbon (Pérez et al., 2006; Hii et al., 2017; Vilasboa et al., 2019). Compared with diploid individuals, polyploid plants usually exhibit superior growth and carbon absorption, which reflects improved photosynthetic efficiency after polyploidization (Liao et al., 2016; Li et al., 2019). Therefore, polyploid *Eucalyptus* is potentially important to improve plant biomass accumulation and to mitigate global warming. Photosynthesis occurs mainly in chloroplasts (Gan et al., 2019). *GLK* family genes are associated with chloroplast development and chlorophyll synthesis in many plant taxa (Chen et al., 2016). However, after whole-genome duplication, the gene dosage effect and epigenetic modification may affect gene expression and ultimately lead to trait variation (Allario et al., 2013; Zhang et al., 2015). For example, in triploid poplar, genes associated with chlorophyll synthesis are upregulated as a result of the gene dosage effect, which lead to increase in chlorophyll content (Du et al., 2020). The effect of *GLK* genes on chloroplast development and chlorophyll synthesis in *Eucalyptus* of different ploidies remains to be studied.

In this study, 40 *GLK* family genes were identified by genome-wide analysis of the genome of *Eucalyptus grandis*. The chromosomal distribution, phylogenetic relationships, conserved motifs, intron and exon structure, and *cis*-acting regulatory elements of *GLK* genes were analyzed. In combination with analysis of the chlorophyll content in *E. urophylla* of different ploidies, the effect of *GLK* family genes on chlorophyll synthesis was studied based on RNA-sequencing data and co-expression network analysis. In addition, putative miRNAs targeting *EgrGLK* genes were also been predicted. The results are important to enhance understanding of the *GLK* gene family and provide a reference for studying the molecular mechanism of the increase in chlorophyll content in polyploid plants.

## Materials and methods

### Identification of *GLK* genes in *Eucalyptus grandis*

The analysis done in this study was presented in Supplementary Figure S1 in the form of flow chart. To identify the *GLK* genes of *E. grandis*, genomic data were downloaded from the Phytozome database.^1^ Using published *GLK* protein sequences from *Arabidopsis*, maize, and tomato as query sequences (Liu et al., 2016; Alam et al., 2022; Wang et al., 2022), the *GLK* protein sequences in the *E. grandis* reference genome were identified with the BLASTP tool (*E*-value: 1e^−5^). The identified sequences were submitted to the SMART online tool^2^ and the NCBI Web CD-Search Tool^3^ for further confirmation of *GLK* proteins. The protein sequences that included a *GLK* domain (PF00249) were retained and designated *EgrGLK*. The physical parameters and subcellular localization of these proteins were predicted with the ExPASy^4^ and WoLF PSORT^5^ online tools.

### Chromosomal location and gene duplication

The location of genes on chromosome and the analysis of gene duplication can provide us with more genetic information about the *EgrGLK* genes. Information on the chromosomal location of each *EgrGLK* gene was extracted from the Phytozome database and the identified *EgrGLK* genes were mapped to individual chromosomes using TBtools (Chen et al., 2020). The duplication landscape of *EgrGLK* genes and cross-species collinearity of *GLK* genes was confirmed with MCScanX software (Wang et al., 2012). The parameters non-synonymous mutations (*K*_a_), synonymous mutations (*K*_s_) and estimated evolutionary constraints (*K*_a_/*K*_s_) among the *EgrGLK* genes were calculated using TBtools (Chen et al., 2020).

### Phylogenetic analysis

To explore the evolutionary relationships of *GLK* proteins in plants, a phylogenetic tree was constructed derived from the *EgrGLK* protein sequences and published *GLK* protein sequences from *Arabidopsis*, maize, and tomato. A multiple sequence alignment of the sequences was generated with ClustalX (Thompson et al., 1997). A phylogenetic tree with 1,000 bootstrap replicates was generated using the neighbor-joining method with MEGAX software (Kumar et al., 2018). The tree was manipulated with the iTOL online tool.^6^

### Analysis of gene structure, conserved motifs, and *cis*-acting regulatory elements

Structure, conserved motifs and *cis*-elements of genes can provide important information for understanding gene function. The structure of the *EgrGLK* genes was analyzed with the GSDS platform^7^ for prediction of introns and exons. The conserved motifs of the *EgrGLK* proteins were predicted using the MEME Suite online tool.^8^ The identified motifs were annotated using the NCBI Web CD-Search Tool. The nucleotide sequence 2000 bp upstream of the start codon for the *EgrGLK* genes was extracted from the *E. grandis* reference genome, and the sequences were submitted to the PlantCARE database^9^ for prediction of *cis*-acting regulatory elements. Conserved motifs, gene structure, and *cis*-element information were visualized using TBtools (Chen et al., 2020).

### Measurement of chlorophyll content

To explore the effect of polyploidization on chlorophyll content in plant leaves, triploid *E. urophylla* obtained by sexual polyploidization and its diploid control were used as materials for measurement of chlorophyll content (Yang et al., 2018). Five clones of triploid and diploid *E. urophylla* were selected. Young leaves at the shoot tips, fully expanded mature leaves, and senescent leaves were randomly selected. The chlorophyll content was determined following the method described by Du et al. (2020). Fresh leaf tissue (1 g), 5 ml of 95% ethanol, and a small amount of quartz sand and calcium carbonate were mixed and ground into a homogenate. An additional 5 ml of 95% ethanol was added and ground further. After standing for 3 min, the homogenate was filtered into a 50 ml brown volumetric flask and diluted to 50 ml with 95% ethanol. Absorbance (*A*) was measured at 645 and 663 nm using a spectrophotometer (Ultrospec 6300 Pro, Biochrom, Cambridge, United Kingdom). The chlorophyll content (mg/g) was calculated with the formula 8.02 × *A*_663_ + 20.20 × *A*_645_.

### Transcriptome analysis

In order to reveal the reasons for the changes of chlorophyll content in plant leaves after polyploidization, terminal buds, young leaves, mature leaves, and senescent leaves were collected from the triploid and diploid *E. urophylla* clones. Total RNA was extracted using the TRIzol Kit (Invitrogen, Carlsbad, CA, United States). The cDNA libraries were prepared using the TruSeq Stranded Total RNA HT Sample Prep Kit (Illumina, San Diego, CA, United States). Following the manufacturer’s recommended protocol, transcriptome sequencing was performed on an Illumina HiSeq 4000 platform by Lc-bio technologies Co., Ltd. (Hangzhou, China). The abundance of transcripts was expressed as reads per kilobase per million mapped reads. The transcriptome data for *EgrGLK* genes was log_2_-transformed. The expression patterns and differential expression among *E. urophylla* clones of different ploidies were visualized by means of a heatmap with TBtools (Chen et al., 2020). In addition, *EgrGLK* genes in leaves were annotated based on the GO database^10^ to understand their functions.

### Co-expression network construction

Co-expression networks were generated to identify which *EgrGLK* genes might play an important role in chlorophyll synthesis. Transcriptome data for genes associated with chlorophyll synthesis and *EgrGLK* genes were subjected to a Pearson correlation analysis. Genes with a Pearson correlation coefficient within the appropriate range (*r* ≥ 0.6 or ≤ −0.6) were selected to generate a co-expression network using Cytoscape software (Kohl et al., 2011).

### qRT-PCR

To determine the reliability of the RNA-seq data, 5 *EgrGLK* genes in leaves were selected for qRT-PCR analysis. Terminal buds, young leaves, mature leaves, and senescent leaves of triploid and diploid *E. urophylla* were used for qRT-PCR analysis. qPCR was subsequently performed using a TransStart® Tip Green qPCR SuperMix (TransGen Biotech, Beijing, China) in 25 μl volume on the 7500 Fast real-time PCR system (Thermo Fisher, Singapore) according to the manufacturer’s instructions. Three technical replicates and three biological replicates were performed on all reactions. The primers and reference gene used for qRT-PCR analysis are listed in Supplementary Table S1.

### Prediction of miRNAs targeting *EgrGLK* genes

To understand the regulation of gene expression at the post-transcriptional level, we predicted the putative miRNAs targeting *EgrGLK* genes. The miRNA sequences of *E. grandis* were downloaded from a previous study, and the miRNAs found in leaves were used for analysis (Lin et al., 2018). The miRNAs targeting *EgrGLK* genes were predicted by submitting the miRNAs and *EgrGLK* genes to psRNATarget.^11^ Cytoscape was used to establish the regulatory network of miRNAs and *EgrGLK* genes (Kohl et al., 2011).

## Results

### Identification and analysis of *GLK* genes in *Eucalyptus grandis*

A total of 40 putative *GLK* proteins were identified in the *E. grandis* genome database (Table 1 and Supplementary Table S2). The online tools NCBI Web CD-Search and SMART were used to verify the identity of the proteins to ensure that they contained conserved *GLK* domains (Supplementary Table S3). The verified proteins were designated *EgrGLK1* to *EgrGLK40*. The molecular weight and isoelectric point of each *EgrGLK* protein are listed in Table 1. The proteins ranged in size from 102 aa (*EgrGLK16*) to 689 aa (*EgrGLK12*). The molecular weight ranged from 11.69 kDa (*EgrGLK16*) to 74.96 kDa (*EgrGLK12*). The isoelectric point ranged from 4.77 (*EgrGLK4*) to 10.22 (*EgrGLK16*). In addition, 38 of the 40 *EgrGLK* proteins were predicted to be localized in the nucleus.

**Table 1:** Physical parameters of *GLK* transcription factors in *Eucalyptus grandis*.

Name	Gene ID	Chromosome	Start	End	PI	Mw (kDa)	Strand	CDS length (bp)	Protein length (aa)	Location
*EgrGLK1*	Eucgr.A00189.1.v2.0	Chr01	7805221	7809813	5.81	72504.39	+	2,007	669	Nuclear
*EgrGLK2*	Eucgr.A01323.1.v2.0	Chr01	8233113	8239446	5.21	53070.55	+	1,455	485	Nuclear
*EgrGLK3*	Eucgr.A01857.1.v2.0	Chr01	33608330	33612765	6.01	50954.1	−	1,404	468	Nuclear
*EgrGLK4*	Eucgr.A01921.1.v2.0	Chr01	34530669	34532062	4.77	19053.88	−	516	172	Nuclear
*EgrGLK5*	Eucgr.A02031.1.v2.0	Chr01	35741939	35746799	5.83	24059.79	+	639	213	Nuclear
*EgrGLK6*	Eucgr.A02082.1.v2.0	Chr01	36277691	36280320	6.82	41747.57	+	1,134	378	Nuclear
*EgrGLK7*	Eucgr.A02201.1.v2.0	Chr01	37382917	37384778	9.25	32763.84	−	930	310	Nuclear
*EgrGLK8*	Eucgr.A02444.1.v2.0	Chr01	39776908	39779403	7.63	42957.85	−	1,155	385	Nuclear
*EgrGLK9*	Eucgr.B02155.1.v2.0	Chr02	40667707	40672664	5.20	40932.61	−	1,116	372	Nuclear
*EgrGLK10*	Eucgr.B02313.1.v2.0	Chr02	42073119	42074948	4.77	37255.93	−	1,011	337	Nuclear
*EgrGLK11*	Eucgr.B02627.1.v2.0	Chr02	46202988	46206837	8.19	31746.74	−	861	287	Nuclear
*EgrGLK12*	Eucgr.C00380.1.v2.0	Chr03	6065170	6069823	5.61	74964.13	−	2,067	689	Nuclear
*EgrGLK13*	Eucgr.C04050.1.v2.0	Chr03	83173778	83177867	7.60	55374.64	+	1,512	504	Nuclear
*EgrGLK14*	Eucgr.C04155.1.v2.0	Chr03	78649962	78652848	6.60	52138.75	+	1,440	480	Nuclear
*EgrGLK15*	Eucgr.D00972.2.v2.0	Chr04	21132122	21137252	6.50	33078.83	+	927	309	Nuclear
*EgrGLK16*	Eucgr.D01346.1.v2.0	Chr04	17250462	17250970	10.22	11689.9	+	306	102	Mitochondrial
*EgrGLK17*	Eucgr.D02225.1.v2.0	Chr04	35765713	35770752	9.33	41990.75	+	1,128	376	Nuclear
*EgrGLK18*	Eucgr.D02611.1.v2.0	Chr04	40020902	40024966	9.04	32064.09	−	882	294	Nuclear
*EgrGLK19*	Eucgr.E00234.1.v2.0	Chr05	2267724	2273201	9.14	43135.71	−	1,167	389	Nuclear
*EgrGLK20*	Eucgr.E00246.1.v2.0	Chr05	2412510	2421762	9.17	38620.75	−	1,041	347	Nuclear
*EgrGLK21*	Eucgr.E00308.1.v2.0	Chr05	2880990	2887358	5.50	62253.87	−	1,674	558	Nuclear
*EgrGLK22*	Eucgr.E02754.1.v2.0	Chr05	48944350	48945391	5.59	21601.73	+	573	191	Nuclear
*EgrGLK23*	Eucgr.E04232.1.v2.0	Chr05	74981964	74988000	5.69	36040.02	+	990	330	Nuclear
*EgrGLK24*	Eucgr.F02896.1.v2.0	Chr06	41128595	41132747	6.70	49407.98	+	1,353	451	Nuclear
*EgrGLK25*	Eucgr.F04055.1.v2.0	Chr06	51329899	51336284	5.62	54973.18	+	1,497	499	Nuclear
*EgrGLK26*	Eucgr.F04475.1.v2.0	Chr06	57276036	57278443	6.59	39694.62	+	1,077	359	Nuclear
*EgrGLK27*	Eucgr.G01503.1.v2.0	Chr07	24195911	24199198	6.48	44709.24	−	1,224	408	Nuclear
*EgrGLK28*	Eucgr.G02094.1.v2.0	Chr07	40710921	40719657	5.99	72986.17	−	2,034	678	Nuclear
*EgrGLK29*	Eucgr.G02343.1.v2.0	Chr07	44100593	44103437	7.04	43255.47	−	1,185	395	Nuclear
*EgrGLK30*	Eucgr.G02494.1.v2.0	Chr07	45799252	45801435	6.57	39241.02	+	1,062	354	Nuclear
*EgrGLK31*	Eucgr.H00055.1.v2.0	Chr08	4287466	4292435	6.08	33816.32	−	942	314	Cytoplasmic
*EgrGLK32*	Eucgr.H01993.1.v2.0	Chr08	20710917	20713708	6.00	48317.01	+	1,278	426	Nuclear
*EgrGLK33*	Eucgr.H04693.1.v2.0	Chr08	65832185	65836257	5.64	64221.54	−	1719	573	Nuclear
*EgrGLK34*	Eucgr.I01178.1.v2.0	Chr09	22723451	22725940	5.59	40274.79	−	1,113	371	Nuclear
*EgrGLK35*	Eucgr.J00182.1.v2.0	Chr10	1909756	1911619	8.59	45164.48	+	1,215	405	Nuclear
*EgrGLK36*	Eucgr.J01904.1.v2.0	Chr10	24412949	24413573	9.37	15323.26	−	411	137	Nuclear
*EgrGLK37*	Eucgr.K01056.1.v2.0	Chr11	13546489	13549812	6.12	48814.58	−	1,380	460	Nuclear
*EgrGLK38*	Eucgr.K01476.1.v2.0	Chr11	18158377	18161567	7.00	45984.74	+	1,260	420	Nuclear
*EgrGLK39*	Eucgr.K01670.1.v2.0	Chr11	19659447	19661493	6.43	32447.42	−	906	302	Nuclear
*EgrGLK40*	Eucgr.K02966.2.v2.0	Chr11	37110402	37113349	6.48	41802.11	+	1,101	367	Nuclear

### Chromosomal location and duplication of *EgrGLK* genes

**Figure 1:**
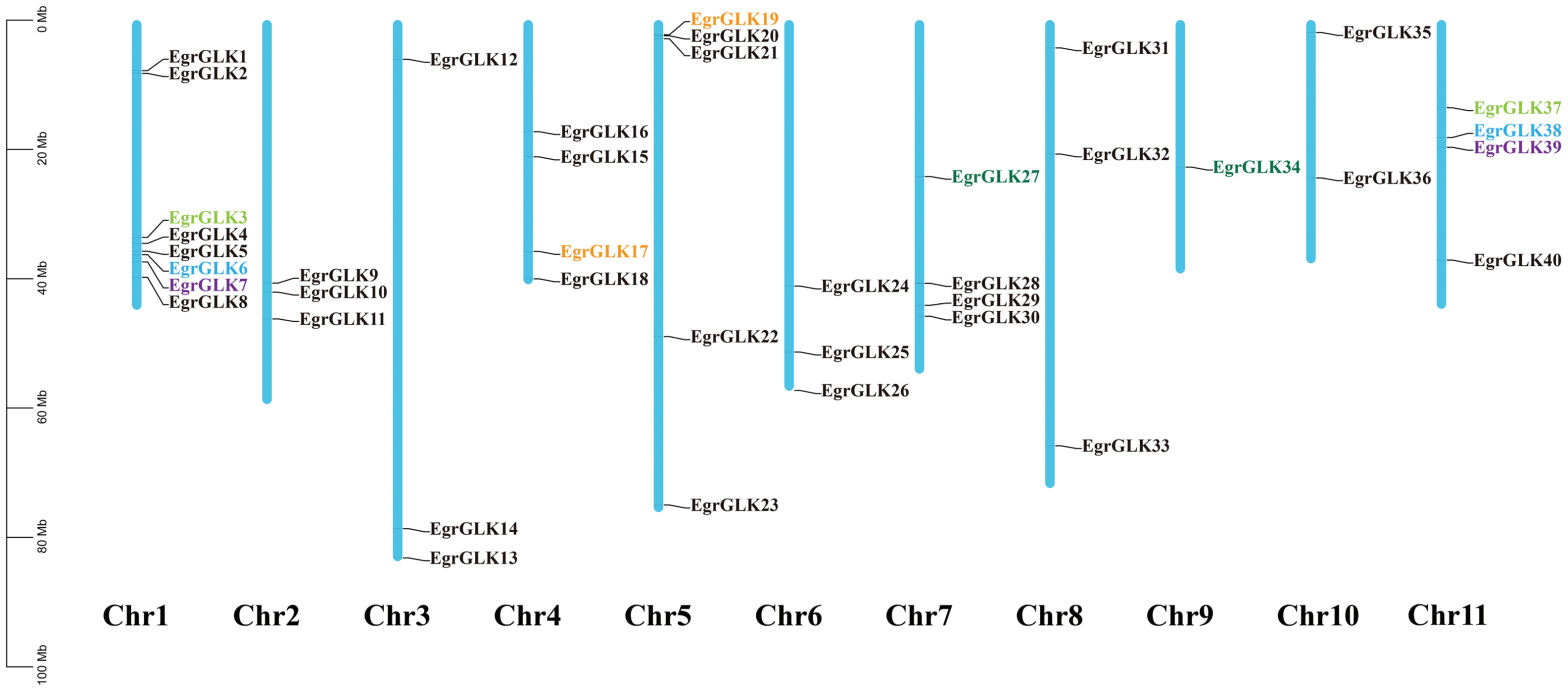
Chromosomal location of *EgrGLK* genes in *Eucalyptus grandis*. Genes of the same color represents a pair of segmented duplicated genes, and genes of different colors represent different gene pairs. No segmental duplication in black genes.

**Figure 2:**
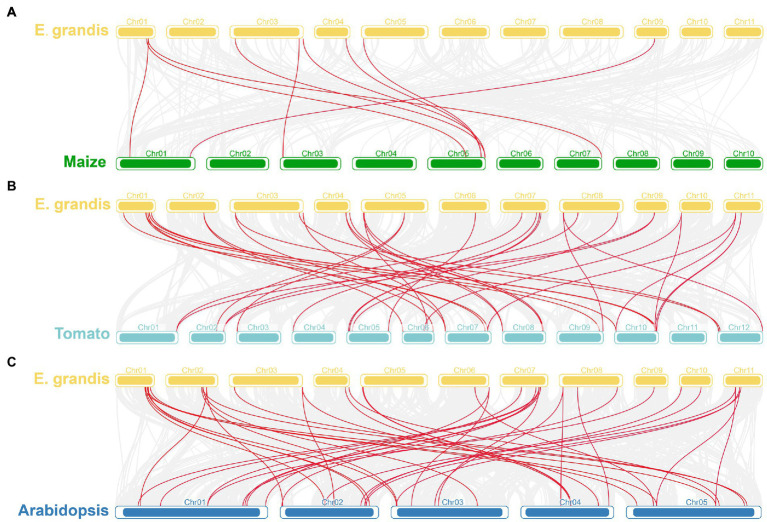
Synteny analysis of *GLK* genes in different plants. Synteny analyses of *GLK* genes between *E. grandis* and maize **(A)**, tomato **(B)**, and *Arabidopsis* **(C)**. Red lines indicate the homologous *GLK* genes between *E. grandis* genome and other plant genomes.

Based on chromosomal position data, the 40 *EgrGLK* genes were mapped to 11 chromosomes (Figure 1). The distribution of *EgrGLK* genes on chromosome was uneven. Chr1 carried eight genes, whereas Chr9 contained only one gene. The longest chromosome, Chr3, contained three genes, only one gene more than the shortest chromosome (Chr10). The number of *EgrGLK* genes on the other chromosomes ranged from three to five. Thus, no correlation between chromosome length and *EgrGLK* gene number was apparent. Investigation of potential duplication events identified five duplicated pairs of *EgrGLK* genes as the products of segmental duplication (Figure 1 and Supplementary Table S4). In addition, the synteny relationships were displayed by comparing the genome of *E. grandis* with those of the other three species (Figure 2). These species include two dicotyledons (*Arabidopsis* and tomato) and one monocotyledon (maize). A total of 41, 39 and 9 homologous gene pairs were identified between *E. grandis* and the three species, respectively. To estimate the type of evolutionary selection on the duplicated *EgrGLK* genes, the *K*_a_, *K*_s_, and *K*_a_/*K*_s_ ratio among the gene pairs were calculated (Supplementary Table S4), which indicated that all gene pairs were subject to purifying selection (*K*_a_/*K*_s_ < 1).

### Phylogenetic relationships of *EgrGLK* proteins

**Figure 3:**
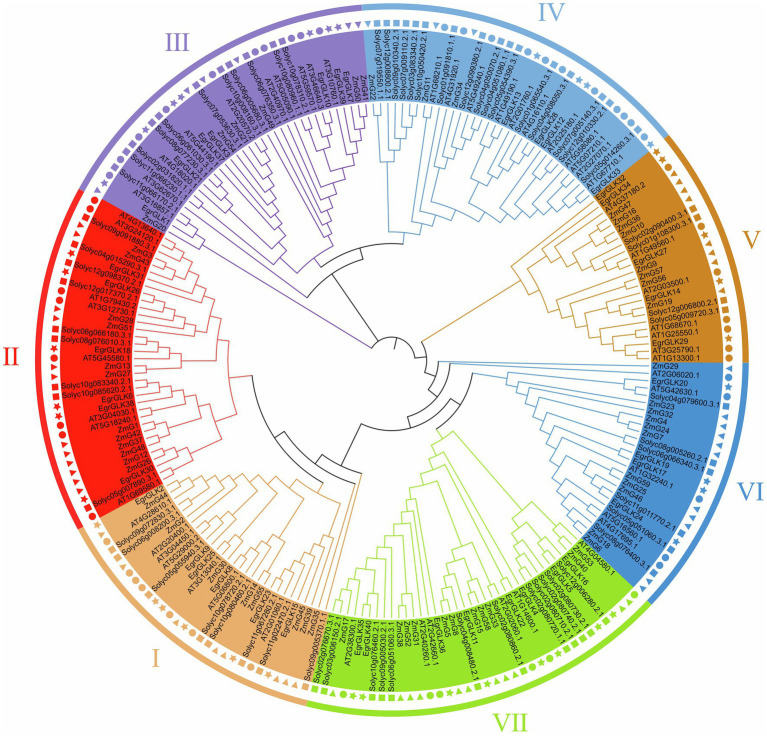
Phylogenetic analysis of *GLK* proteins from *E. grandis*, maize, *Arabidopsis*, and tomato. Different *GLK* protein groups are indicated by different colors. The star, triangle, circle, and square represent *E. grandis*, maize, *Arabidopsis*, and tomato *GLK* proteins, respectively.

To explore the evolutionary relationships of *GLK* proteins in plants and classify the identified *EgrGLK* proteins, a neighbor-joining tree was constructed. The *EgrGLK* proteins were clustered into seven groups based on their grouping with *Arabidopsis*, maize, and tomato *GLK* proteins (Figure 3). The *GLK* proteins from all four species were clustered in each group, but the 40 *EgrGLK* proteins were unevenly distributed. Eight *EgrGLK* proteins were clustered in group VII, which was double the number of *EgrGLK* proteins in groups IV and VI. Six, six, seven, and five *EgrGLK* proteins were included in groups I, II, III, and V, respectively. *EgrGLK* proteins were distributed in each group, which provided information on the orthologous relationships and strong evolutionary conservation among *GLK* proteins of different species.

### Analysis of gene structure and conserved motifs of *EgrGLK* genes

**Figure 4:**
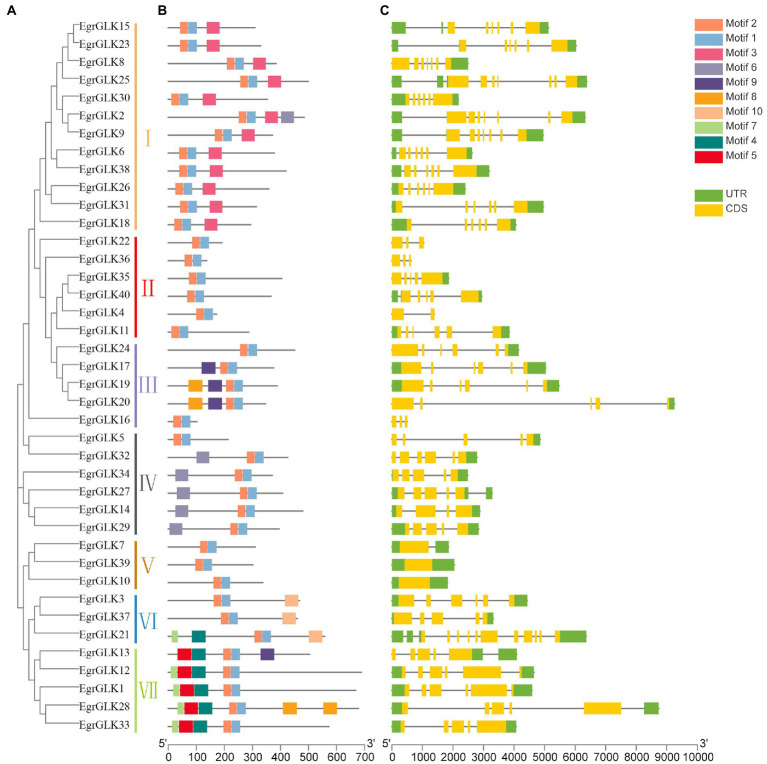
Phylogenetic tree, conserved motifs, and gene structure of *EgrGLK* genes of *E. grandis*. **(A)** Phylogenetic reconstruction for *EgrGLK* proteins. **(B)** Ten conserved motifs indicated by different colors. **(C)** Gene structure with exons and introns indicated.

To further explore evolutionary relationships among the *EgrGLK* genes, a phylogenetic tree was generated for the 40 *EgrGLK* protein sequences. The proteins were resolved into seven groups (Figure 4A). The conserved motifs of the proteins were analyzed using the online MEME tool, and the conserved sequences of each motif are shown in Supplementary Table S5. Seven putative motifs were functionally annotated, which were defined as MYB-SHAQKYF for motif 1, components of the conserved *GLK* domain for motifs 2 and 10, MYB-CC-LHEQLE for motif 3, and the REC superfamily for motifs 4, 5, and 7. No functional annotation was assigned for the remaining three putative motifs (Figure 4B). The MYB-SHAQKYF motif was observed to be a component of the conserved *GLK* domain. All *EgrGLK* proteins contained motifs 1 and 2, which indicated that these two motifs constituted the basic *GLK* domain associated with the typical function. The proteins contained different motifs in accordance with the phylogenetic grouping. Motif 3 was only detected in group I, motifs 4, 5, and 7 were coincident in group VII, and motifs 6 and 9 were mostly present in groups IV and III, respectively. Motif 8 was only detected in *EgrGLK19*, *EgrGLK20*, and *EgrGLK28*. Motif 10 was only identified in group VI, which was the only group to contain three conserved *GLK* motifs. In general, proteins in the same group contained basically the same conserved motifs, indicating that these proteins perform similar functions within a group.

To examine the structural variation among the *EgrGLK* genes, the exon–intron organization of each *EgrGLK* gene was assessed based on the phylogenetic classification (Figure 4C). Differences in the number of introns between genes were observed in different groups. No introns were detected in group V, and the gene (*EgrGLK21*) with the most introns was classified in group VI. In group II, the intron number ranged from one to five. In addition, most *EgrGLK* genes that were clustered in the same phylogenetic group showed similar exon–intron structures.

### Analysis of *cis*-regulatory elements in the promoter regions of *EgrGLK* genes

**Figure 5:**
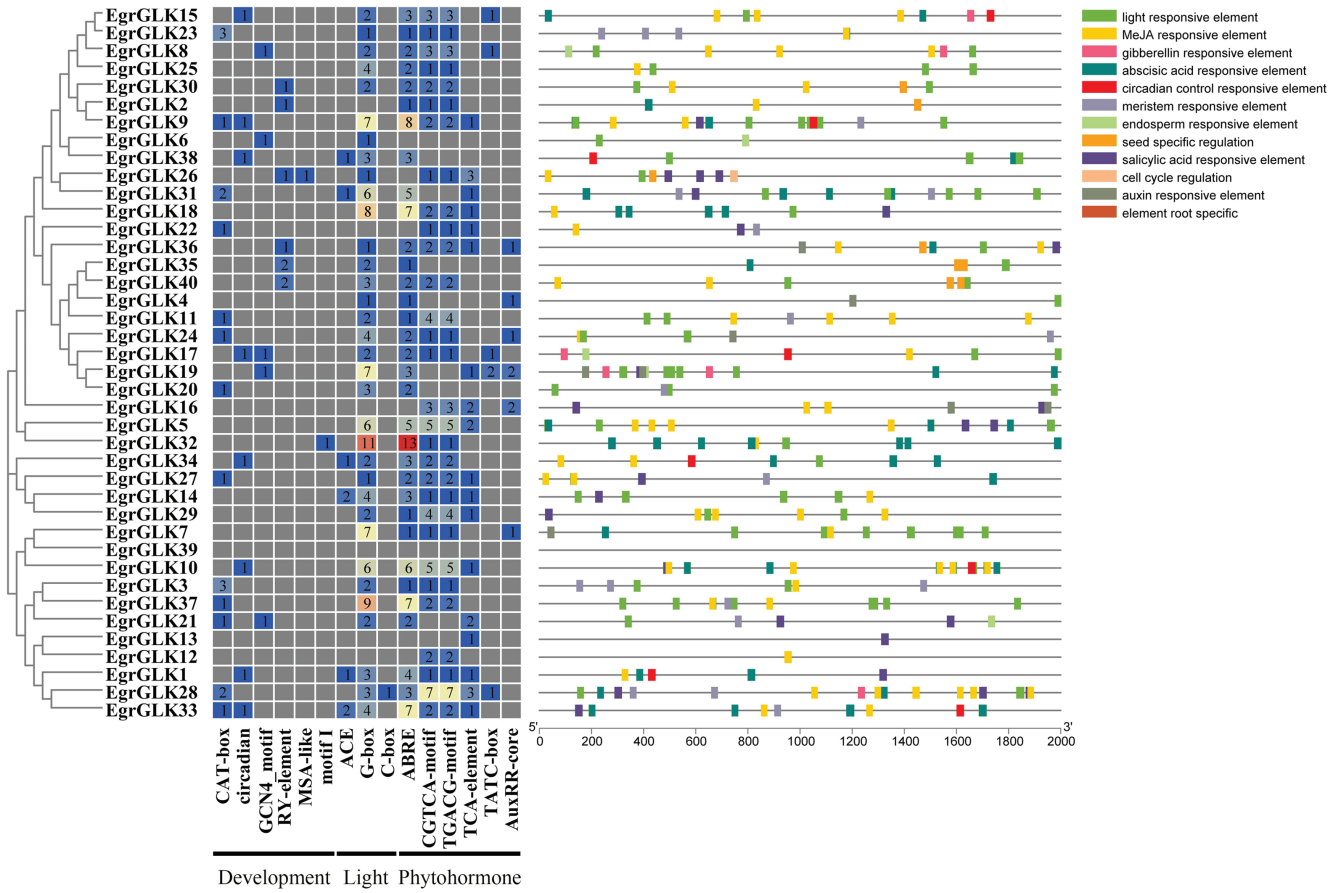
Analysis of *cis*-acting regulatory element in promoter regions of *EgrGLK* genes of *E. grandis*. Numerals in different color boxes indicate the number of the main *cis*-elements of *EgrGLK* genes. *Cis*-elements of different responsive types are indicated by different colors.

The presence of *cis*-acting regulatory elements in promoter regions is important for the expression of downstream target genes and the regulation of transcription factor interaction. Therefore, *cis*-regulatory elements related to development, light response, and hormone response in the promoter regions of the *EgrGLK* genes were investigated (Supplementary Table S6 and Figure 5). The most abundant putative *cis*-elements were involved in hormone response and comprised ABRE, CGTCA-motif, TGACG-motif, TCA-element, TATC-box, and AuxRR-core elements. The ABRE elements were distributed in the promoter regions of 33 *EgrGLK* genes and are involved in abscisic acid response. The CGTCA-motif and TGACG-motif are involved in methyl jasmonate response; thus, *cis*-regulatory elements responsive to methyl jasmonate were the most frequent. Three types of light-responsive elements were identified, namely ACE, G-box, and C-box. Among all *cis*-regulatory elements identified, G-box elements were the most widely distributed and were identified in 34 *EgrGLK* genes, which indicated that many *EgrGLK* genes may be sensitive to light. In addition, five *cis*-elements involved in development were identified, comprising CAT-box, circadian, GCN4-motif, RY-element, MSA-like elements and motif I elements. These elements are involved in plant growth, cell division, and diverse plant-specific tissues. Notably, many *cis*-regulatory elements consisted of two or more copies in the 2 kb upstream region, which may enhance their binding to the corresponding transcription factors.

### Leaf chlorophyll content of diploid and triploid *Eucalyptus urophylla*

**Figure 6:**
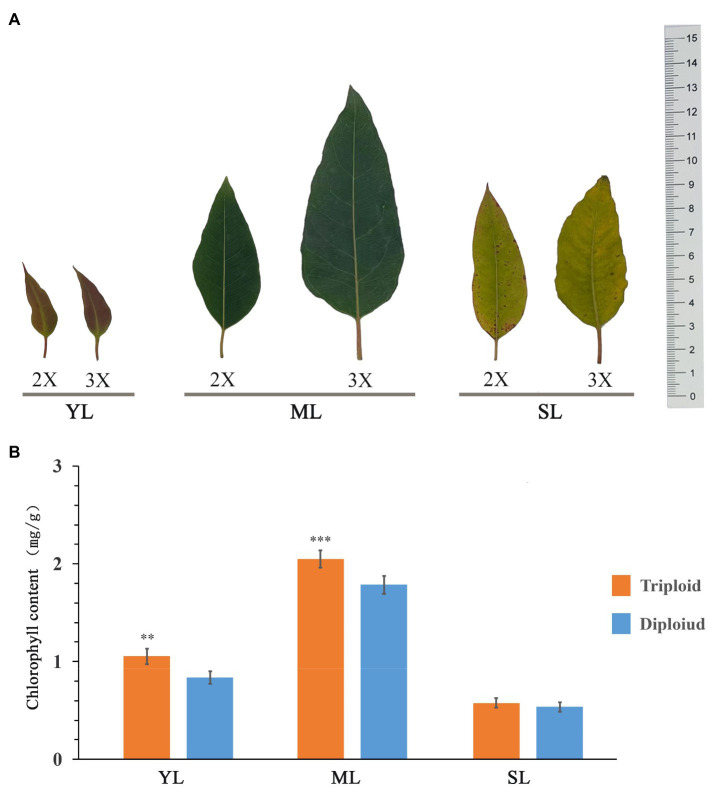
Measurement of chlorophyll content in leaves of *E. urophylla*. Phenotype **(A)** and chlorophyll content **(B)** of young leaves (YL), mature leaves (ML), and senescent leaves (SL) of diploid (2X) and triploid (3X) clones.

Triploid *E. urophylla* and its diploid control were used to measure the chlorophyll content in young, mature, and senescent leaves (Figure 6). The trend in chlorophyll content of the different leaves of the diploid and triploid clones was identical. With increase in leaf age, the chlorophyll content initially increased and then decreased, thus the chlorophyll content was highest in mature leaves. The leaf chlorophyll content was higher in the triploid than in the diploid, and in young and mature leaves the chlorophyll content of the triploid was significantly higher than that of the diploid.

### Expression analysis of *EgrGLK* genes in leaves of diploid and triploid *Eucalyptus urophylla*

To explore the influence of *EgrGLK* gene expression on chlorophyll synthesis in diploid and triploid *E. urophylla*, transcriptome data from leaves of four developmental stages (terminal buds, young leaves, mature leaves, and senescent leaves) were used to analyze the transcript abundance. The transcripts of 36 *EgrGLK* genes were detected in the leaves. Then, the GO term classification and enrichment analysis of 36 *EgrGLK* genes were performed. The first twenty GO terms were shown in the Supplementary Figure S2 according to the significance of enrichment. And the most enriched five GO terms were ‘regulation of transcription’, ‘DNA-binding transcription factor activity’, ‘transcription’, ‘DNA binding’ and ‘nucleus’. Among them, there were 35 and 33 *EgrGLK* genes involved in ‘nucleus’ and ‘regulation of transcription’, respectively, indicating that *EgrGLK* genes mainly functions as transcription factors in the nucleus.

**Figure 7:**
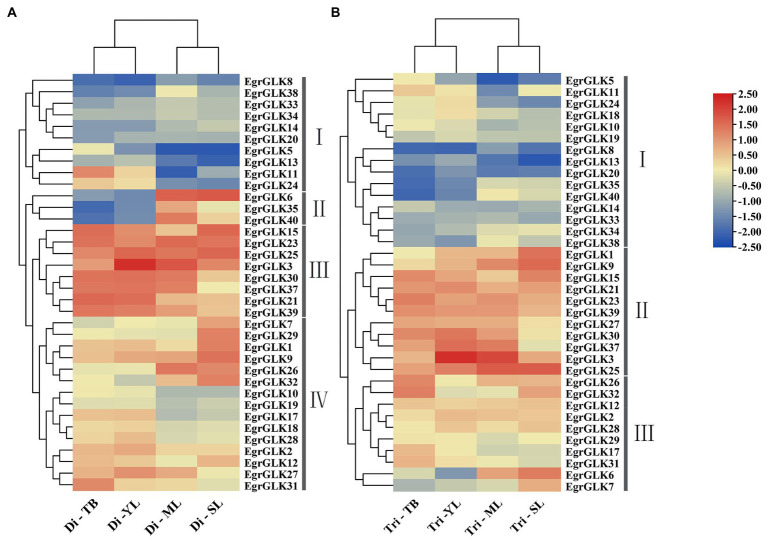
Expressions patterns of *EgrGLK* genes in leaves of *E. urophylla*. The expression patterns of *EgrGLK* genes in the terminal bud (TB), young leaves (YL), mature leaves (ML), and senescent leaves (SL) of diploid **(A)** and triploid **(B)** clones.

The expression data for these 36 *EgrGLK* genes were log_2_-transformed and used to generate a clustered heatmap to visualize the expression patterns at each leaf developmental stage (Figure 7). The 36 *EgrGLK* genes were divided into four and three groups in diploid and triploid *E. urophylla*, respectively. Ten *EgrGLK* genes were included in group I of the diploid and 15 *EgrGLK* genes were included in group I of the triploid, which exhibited low transcript levels at each developmental stage. Group IV of the diploid and group III of the triploid contained 15 and 10 *EgrGLK* genes, respectively, and exhibited relatively high transcription levels. Group III of the diploid and group II of the triploid contained eight and 11 *EgrGLK* genes, respectively, and these genes exhibited high transcript abundance in all analyzed leaves, hinting that these genes were essential in *E. urophylla* leaves. It was noteworthy that *EgrGLK6*, *EgrGLK35*, and *EgrGLK40* were grouped in group II of the diploid. These three genes showed low transcript abundance in terminal buds and young leaves, and high expression levels in mature and senescent leaves. The same expression patterns of *EgrGLK6*, *EgrGLK35*, and *EgrGLK40* were observed in leaves of the triploid; however, the differential expression of these three *EgrGLK* genes in the triploid was more moderate than in the diploid, hence they were not clustered into a separate group. In general, the expression patterns of most *EgrGLK* genes in leaves of the diploid and triploid were approximately identical.

### Differential expression analysis of *EgrGLK* genes in leaves of diploid and triploid *Eucalyptus urophylla*

**Figure 8:**
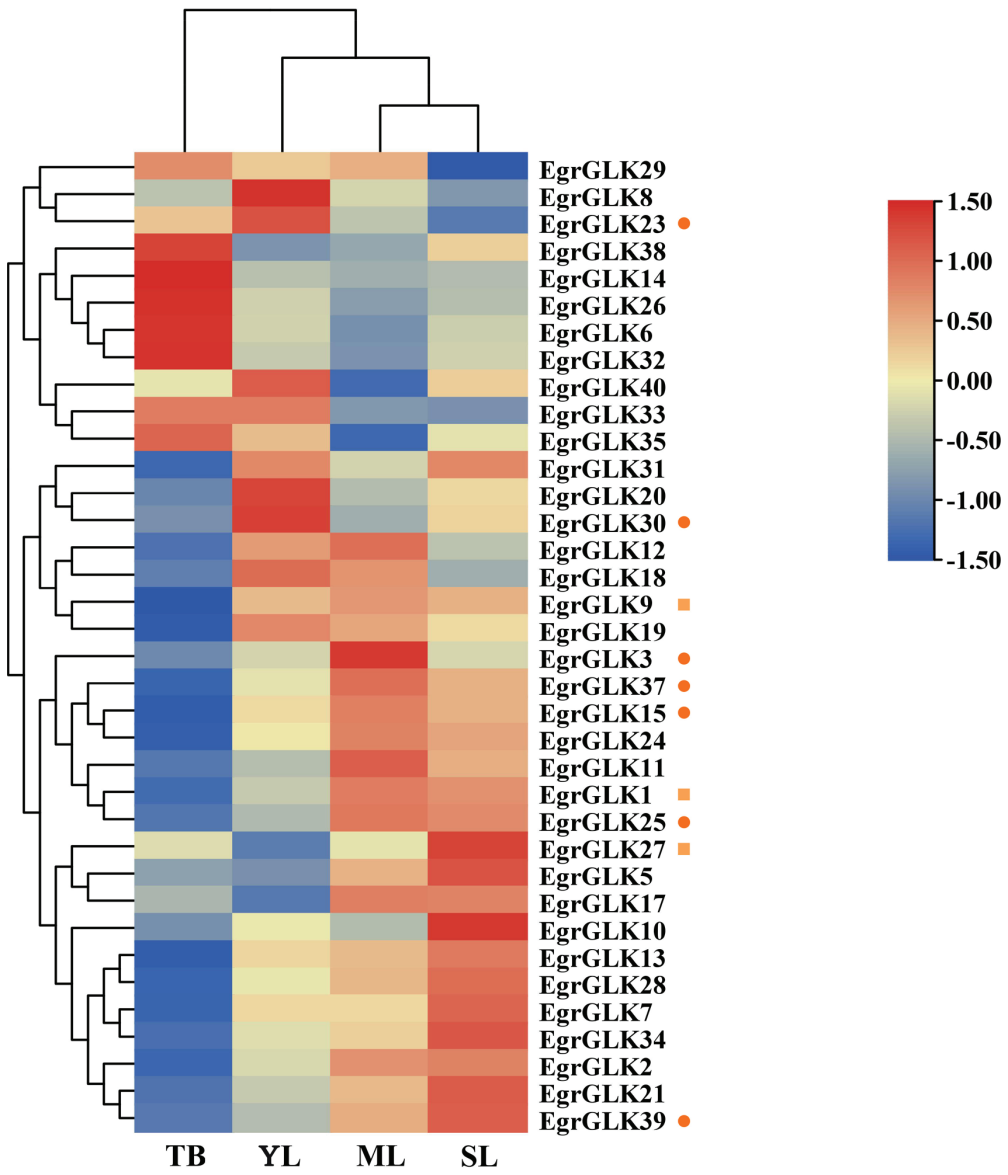
Differential expression of *EgrGLK* genes in the terminal bud (TB), young leaves (YL), mature leaves (ML), and senescent leaves (SL) of diploid and triploid clones. Circles represent highly expressed genes common to the diploid and triploid clones, and squares represent genes highly expressed only in the triploid clone.

To further explore the effect of differential expression of *EgrGLK* genes on chlorophyll synthesis in leaves of *E. urophylla* of different ploidies, the expression data for the *EgrGLK* genes in diploid *E. urophylla* leaves were used as the control group, and the fold change in expression between triploid leaves and diploid leaves was used to generate heat maps (Figure 8). More than half of the *EgrGLK* genes were down-regulated in terminal buds of triploid *E. urophylla*. In contrast, 30, 24, and 28 *EgrGLK* genes were upregulated in young, mature, and senescent leaves of triploid *E. urophylla* (Supplementary Table S7). Among the genes highly expressed in leaves that were common to diploid and triploid *E. urophylla* (Figure 7, *EgrGLK23*, *EgrGLK30*, *EgrGLK3*, *EgrGLK37*, *EgrGLK15*, *EgrGLK25*, and *EgrGLK39*), *EgrGLK3*, *EgrGLK15*, *EgrGLK25*, *EgrGLK37* and *EgrGLK39* were upregulated in triploid young (1.03, 1.05, 1.10, 1.01 and 1.04 fold change, respectively), mature (1.77, 1.22, 2.03, 1.48 and 1.46 fold change, respectively), and senescent leaves (1.04, 1.13, 1.91, 1.24 and 1.84 fold change, respectively), and down-regulated in triploid terminal buds (Figure 8). Three genes were highly expressed only in the triploid, of which *EgrGLK1* and *EgrGLK9* showed the same differential expression pattern as the common highly expressed genes, and the other gene (*EgrGLK27*) was upregulated in all analyzed leaves. To further confirm the effect of polyploidization on gene expression, genes upregulated in triploid including *EgrGLK3*, *EgrGLK15*, *EgrGLK25*, *EgrGLK37* and *EgrGLK39* were selected for qRT-PCR analysis (Supplementary Figure S3). Correlation analysis showed that there was a high correlation coefficient between qRT-PCR and RNA-seq (*R*^2^ = 0.826, *p* < 0.01), indicating that the differential expression of *EgrGLK* genes among different ploidies was reliable. Differential expression of *EgrGLK* genes provided preliminary information for the study of chlorophyll synthesis and leaf development of *E. urophylla* with different ploidies.

### Co-expression network analysis

**Figure 9:**
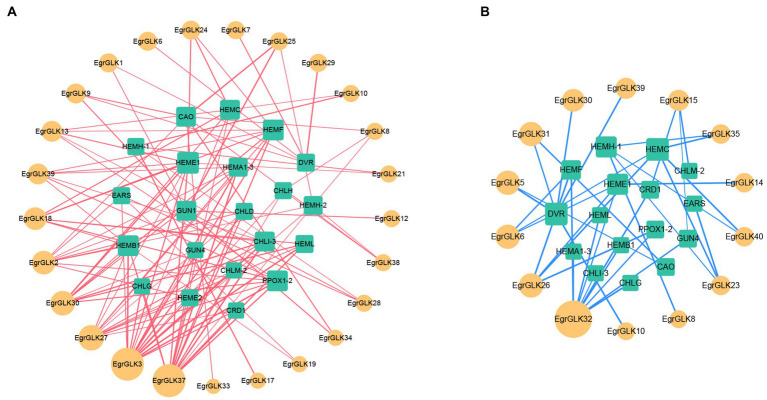
Co-expression network of *EgrGLK* genes and chlorophyll synthesis related genes. Positive correlation network **(A)** and negative correlation network **(B)** between *EgrGLK* genes and chlorophyll synthesis related genes. Circles represent *EgrGLK* genes, squares represent chlorophyll synthesis related genes, and the size of the circle or square represents the number of relationships between genes.

Transcriptome data for genes associated with chlorophyll synthesis and the *EgrGLK* genes were used for a correlation analysis to explore the role of *EgrGLK* genes in chlorophyll synthesis (Supplementary Table S8). The transcriptome data for chlorophyll synthesis related genes and *EgrGLK* genes that were highly correlated (*r* ≥ 0.6 or ≤ −0.6) were used to generate co-expression networks (Figure 9). Twenty-five *EgrGLK* genes and 21 chlorophyll synthesis related genes were involved in the positive correlation co-expression network, forming a total of 115 correlation network lines. Among these genes, *EgrGLK3* and *EgrGLK37* were correlated with 17 and 16 chlorophyll synthesis related genes, respectively. The negative correlation co-expression network incorporated 14 *EgrGLK* genes and 16 chlorophyll synthesis related genes, forming a total of 36 correlation network lines. Among these genes, *EgrGLK32* was correlated to 10 chlorophyll synthesis related genes. These results showed that *EgrGLK* genes and chlorophyll synthesis related genes were mainly positively correlated, and thus that *EgrGLK* genes may play a positive regulatory role in chlorophyll synthesis.

### Prediction of miRNAs targeting *GLK* genes

**Figure 10:**
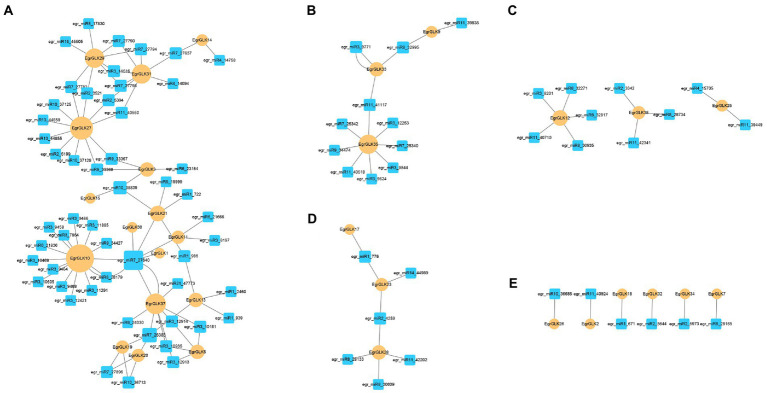
Regulatory networks were divided into **(A-E)** according to the number of targeting relationship between miRNAs and *EgrGLK* genes.

In plants, microRNAs (miRNAs) have been found to play important roles in the regulation of gene expression at the post-transcriptional level. We predicted the miRNAs targeting *EgrGLK* genes to further reveal the possible reasons for the differential expression of *EgrGLK* genes in different ploidies. In previous studies, 179 miRNAs were found in leaves (Lin et al., 2018), of which 85 miRNAs from 23 families were involved in the regulation of 31 *EgrGLK* genes (Supplementary Table S9). According to the targeting relationship between miRNAs and *EgrGLK* genes, 12 regulatory networks were generated and they were divided into 5 groups (Figure 10). Figure 10A showed the most complex regulatory network containing 52 miRNAs and 16 *EgrGLK* genes, while six miRNAs in Figure 10E showed the simplest regulatory network in the type of one-to-one target gene. *EgrGLK3*, *EgrGLK32* and *EgrGLK37* has been identified as the genes that play an important role in chlorophyll synthesis, and there were nine, four and one miRNAs targeting these three genes, respectively. In addition, five *EgrGLK* genes including *EgrGLK10*, *EgrGLK27*, *EgrGLK35*, *EgrGLK29*, *EgrGLK31* with the most targeting relationships were found and they were regulated by 15, 11, 8, 8 and 8 miRNAs, respectively. These results suggest that miRNAs play an important role in the regulation of *EgrGLK* gene expression.

## Discussion

In this study, 40 *EgrGLK* genes were identified in the *E. grandis* genome (Supplementary Table S2). The number of *EgrGLK* genes was less than the number of *GLK* genes identified in Arabidopsis (55), maize (59), and tomato (66) (Liu et al., 2016; Alam et al., 2022; Wang et al., 2022). It was suspected that some *EgrGLK* genes were lost during evolution (Du et al., 2022). It was observed that the number of *GLK* family members was independent of genome size. The diversity of *GLK* family members in different species may be influenced by genome duplication events, such as whole-genome duplication, segmental duplication, or tandem duplication (Zhang, 2003; Chang and Duda., 2012). In the present study, five segmental duplication gene pairs were identified among the *EgrGLK* genes, indicating that segmental duplication was the main contributor to expansion of *GLK* genes in *E. grandis* (Figure 1). It has previously been reported that segmental duplication is more common than tandem duplication in the *GLK* gene family, thus the former might play an important role in chloroplast evolution (Song et al., 2016; Wu et al., 2016). The synteny relationships of *GLK* genes between *E. grandis* and other species showed that the number of homologous gene pairs between *E. grandis* and dicotyledons was much more than that between *E. grandis* and monocotyledons, indicating that *GLK* gene family has been amplified after differentiation between dicotyledon and monocotyledon (Figure 2). The *EgrGLK* genes were classified into seven groups based on a phylogenetic analysis (Figure 3), which was consistent with the classification of *GLK* genes in Arabidopsis (Alam et al., 2022). This result indicated that, although some *EgrGLK* genes were lost during evolution, the sufficient genetic diversity has been retained in *E. grandis*.

As previously reported, the structural characteristics of gene families may reflect their evolutionary trends (Haas et al., 2008; Nie et al., 2022), whereas the conserved motifs reflect their protein-specific functions (Lai et al., 1998; Yang et al., 2021). The 40 *EgrGLK* genes were divided into seven groups, and the gene structure and motif arrangement of the genes in the same group were similar (Figure 4). This finding indicated that the *EgrGLK* genes in the same group might have similar functions (Li et al., 2022; Liang et al., 2022). In the current study, seven motifs were functionally annotated using the NCBI Web CD-Search Tool, which have also been detected in the *GLK* family members in tobacco (Qin et al., 2021). Thus, excluding the *GLK* motifs, other motifs detected in the *GLK* proteins were also relatively conserved. Moreover, group VI was the only group that contained three conserved *GLK* motifs; this phenomenon may enhance the regulatory role of these three *EgrGLK* genes as transcription factors.

The *cis*-acting elements may be activated by *trans*-acting elements to regulate the activity of the target genes (Valli et al., 2022). Among the *cis*-acting elements detected in the promoter regions of *EgrGLK* genes (Figure 5), elements associated with development were only sporadically distributed in the promoter regions, whereas elements involved in light response and hormone response were observed in the promoter regions of almost all *EgrGLK* genes. The motifs involved in methyl jasmonate response comprised the CGTCA-motif and TGACG-motif, which were the most frequent *cis*-regulatory elements identified. These motifs play important roles in multiple physiological processes, including development, senescence, and response to diverse environmental stresses (Browse and Howe, 2008; Wang et al., 2011). The G-box was indicated to be the most widely distributed element. The G-box element may be unique to light regulation and is a ubiquitous element in functionally diverse genes (Menkens et al., 1995; Waters et al., 2009). These results suggested that *EgrGLK* genes are controlled by light and phytohormones (Nakamura et al., 2009; Lupi et al., 2019). The diversity of types, quantity, and distribution of *cis*-acting elements in the gene promoters reflects the complex response regulatory mechanism and complex evolutionary relationships of the *EgrGLK* genes.

The *GLK* genes play an important role in chloroplast development and chlorophyll synthesis (Fitter et al., 2002; Chen et al., 2016). The expression pattern of *GLK* family genes in *E. urophylla* leaves that differ in chlorophyll content was studied to explore the effect of the genes on chlorophyll synthesis. The chlorophyll content in young and mature leaves of triploid *E. urophylla* was significantly higher than that of the diploid clone. Such an increase in chlorophyll content has been observed in other polyploid plants, such as *Populus* and *Rhododendron fortunei* (Liao et al., 2016; Mo et al., 2020). Thirty-six *EgrGLK* genes were expressed in leaves of both diploid and triploid *E. urophylla*. The *EgrGLK* genes not expressed in the leaves might be tissue-specific genes (Deveaux et al., 2003; Lim et al., 2012). The 36 *EgrGLK* genes in the diploid and triploid clones were divided into four and three groups, respectively, based on transcriptome data. The genes *EgrGLK6*, *EgrGLK35*, and *EgrGLK40* in leaves of the diploid were clustered in group II, but did not form a separate group in the triploid (Figure 7). The promoter regions of these *EgrGLK* genes in group II of the diploid contained *cis*-acting elements associated with the seed and endosperm (Figure 5). Triploids are characterized by sterility and thus whether the differential expression pattern of these three genes is associated with triploid sterility requires further study (Fujiwara and Beachy, 1994). Except for group II, other groups in the diploid showed the same expression pattern as in the triploid, which could be divided into low, relatively high, or high expression levels based on the expression data (Figure 7). Compared with the diploid, three additional genes in the triploid were clustered into the high-expression-level group. The high expression levels of a greater number of genes in polyploids might be caused by the participation of duplicated genes derived from whole-genome duplication (Jackson and Chen, 2010; Roulin et al., 2013). The high expression levels of *EgrGLK1*, *EgrGLK9*, and *EgrGLK27* in the triploid may be one factor that promotes the increase in chlorophyll content in leaves of triploid *E. urophylla*.

The differential expression of *GLK* genes in leaves also affects chloroplast development and chlorophyll synthesis (Nguyen et al., 2014). In Arabidopsis, overexpression of *GLK* genes increases the chlorophyll content in leaves (Waters et al., 2009). In the present study, *EgrGLK* genes were differentially expressed in triploid and diploid *E. urophylla* leaves (Figure 8). In the terminal bud, most *EgrGLK* genes in the triploid were down-regulated compared with the diploid, whereas in young, mature, and senescent leaves, more than two-thirds of the *EgrGLK* genes were up-regulated in the triploid. The down-regulation of *EgrGLK* genes in the terminal bud of the triploid did not affect the chlorophyll content of mature leaves, and the chlorophyll content of mature leaves was significantly higher in the triploid than in the diploid (Figure 6). The chlorophyll content in leaves increases gradually with increase in leaf age before the leaves are fully developed and attain maturity (Bertamini and Nedunchezhian, 2002; Du et al., 2020). This is consistent with the present results for *E. urophylla*, indicating that the chlorophyll content might be associated with the expression of *EgrGLK* genes during leaf development. The chlorophyll content of young, mature, and senescent leaves of the triploid was higher than that of diploid. The differential expression of *EgrGLK* genes in leaves of *E. urophylla* of different ploidies was consistent with the trend in chlorophyll content. To further verify the relationship between *EgrGLK* gene expression and chlorophyll synthesis, co-expression networks of the *EgrGLK* genes and chlorophyll synthesis related genes were generated (Figure 9). The networks indicated that *EgrGLK* genes may positively regulate chlorophyll synthesis (Waters et al., 2008; Brand et al., 2014; Zubo et al., 2018). In addition, based on the number of correlations between *EgrGLK* genes and chlorophyll synthesis related genes, three important *EgrGLK* genes were identified. *EgrGLK37* and *EgrGLK3* were involved in the positive correlation network and *EgrGLK32* was included in the negative correlation network (Figure 9). Among these genes, *EgrGLK37* and *EgrGLK3* showed high expression levels in leaves, whereas *EgrGLK32* showed relatively high expression levels in leaves (Figure 7). The present results provide a reference for further studies of the relationship between *EgrGLK* genes and chlorophyll synthesis.

Mature miRNA combines with the RNA-induced silencing complex, which interacts with target genes to regulate the expression of genes by inhibiting gene translation or degrading targeted mRNAs (Baulcombe, 2004). In this study, 85 miRNAs targeting *EgrGLK* genes were predicted, which is almost half of the miRNAs in leaves found in previous study (Lin et al., 2018). *EgrGLK10*, *EgrGLK27*, *EgrGLK29*, *EgrGLK31*, *EgrGLK35 and EgrGLK37* are all regulated by more than eight miRNAs, of which five *EgrGLK* genes do not belong to the group with high transcription level (Figure 7). The result confirmed the inhibitory effect of miRNA on gene expression (Pappas Mde et al., 2015; Unnikrishnan and Shankaranarayana, 2020). *EgrGLK37* is a gene regulated by nine miRNAs but with high expression level. And it is speculated that *EgrGLK37* may also be affected by other regulatory mechanisms, such as lncRNA (Lin et al., 2019; Chen et al., 2021). In addition, *EgrGLK37* was also found to be associated with the most chlorophyll related genes in the co-expression network, indicating that *EgrGLK37* may play an important role in the gene expression regulation network. In this study, three types of miRNAs were found, including one to multiple target genes, one to one target gene and multiple miRNAs to a common target gene, which is consistent with the results found in previous miRNA studies (Lin et al., 2018). Differential expression of *EgrGLK* genes among different ploidies have been proved in our study and the regulation of miRNA may also be one of the reasons for the change of gene expression after plant polyploidization.

## Conclusion

In this study, *GLK* transcription factors of *E. grandis* were systematically analyzed using bioinformatic methods. Forty *EgrGLK* genes were identified in the *E. grandis* genome and were divided into seven groups according to the gene structure and motif composition. The number of *EgrGLK* genes is less than the number of *GLK* genes identified in other species, but the sufficient genetic diversity has been retained in *E. grandis*, which indicates that *GLK* proteins exhibit strong evolutionary conservation across diverse species. Analysis of phenotypic and transcriptome data for leaves at different developmental stages in diploid and triploid *E. urophylla* revealed a positive correlation between *EgrGLK* genes and chlorophyll synthesis. On the basis of a differential expression analysis, it was speculated that the increase in chlorophyll content in leaves of triploid *E. urophylla* may be caused by up-regulation of *EgrGLK* gene expression. In addition, three *EgrGLK* genes that may play an important role in chlorophyll synthesis were identified. The present research provides valuable information for further functional characterization of *EgrGLK* genes in *Eucalyptus*. In the future, increasing the expression of *GLK* gene in plants by polyploidy or other methods may promote photosynthesis and growth of plants, which is of great value to improve plant yield.

## Data availability statement

The data presented in the study are deposited in the GEO repository, accession number GSE207860.

## Author contributions

JY and XK conceived and designed the research. ZL, TX, and YZ conducted the experiments. ZL, BQ, and HC collected and analyzed the data. ZL and JY wrote the manuscript. XK provided the valuable suggestions on the manuscript. All authors contributed to the article and approved the submitted version.

## Funding

This research was supported by the National Natural Science Foundation of China (31901337) and the National Key R&D Program of China during the 14th Five-year Plan Period (2021YFD2200105).

## Conflict of interest

The authors declare that the research was conducted in the absence of any commercial or financial relationships that could be construed as a potential conflict of interest.

## Publisher’s note

All claims expressed in this article are solely those of the authors and do not necessarily represent those of their affiliated organizations, or those of the publisher, the editors and the reviewers. Any product that may be evaluated in this article, or claim that may be made by its manufacturer, is not guaranteed or endorsed by the publisher.

## Footnotes

^1^ https://phytozome-next.jgi.doe.gov

^2^ http://smart.embl-heidelberg.de

^3^ https://www.ncbi.nlm.nih.gov/Structure/bwrpsb/bwrpsb.cgi

^4^ https://web.expasy.org/compute_pi

^5^ https://www.genscript.com/wolf-psort.html

^6^ http://itol.embl.de/

^7^ http://gsds.gao-lab.org/

^8^ http://meme-suite.org/meme/

^9^ http://bioinformatics.psb.ugent.be/webtools/plantcare/html/

^10^ http://www.geneontology.org

^11^ http://plantgrn.noble.org/psRNATarget

## Supplementary material

The Supplementary materials for this article can be found online at: https://www.frontiersin.org/articles/10.3389/fpls.2022.952877/full#supplementary-material

## References

[ref1] Alam I. Wu X. Yu Q. Ge L. (2022). Comprehensive genomic analysis of G2-Like transcription factor genes and their role in development and abiotic stresses in *Arabidopsis*. Diversity 14, 228. doi: 10.3390/d14030228

[ref2] Allario T. Brumos J. Colmenero-Flores J. M. Iglesias D. J. Pina J. A. Navarro L.. (2013). Tetraploid Rangpur lime rootstock increases drought tolerance via enhanced constitutive root abscisic acid production. Plant Cell Environ. 36, 856–868. doi: 10.1111/pce.12021, PMID: 23050986

[ref3] Baulcombe D. (2004). RNA silencing in plants. Nature 431, 356–363. doi: 10.1038/nature02874 15372043

[ref4] Bertamini M. Nedunchezhian N. (2002). Leaf age effects on chlorophyll, Rubisco, photosynthetic electron transport activities and thylakoid membrane protein in field grown grapevine leaves. J. Plant Physiol. 159, 799–803. doi: 10.1078/0176-1617-0597

[ref5] Bhutia K. L. Nongbri E. L. Gympad E. Rai M. Tyagi W. (2020). In silico characterization, and expression analysis of rice Golden 2-Like (OsGLK) members in response to low phosphorous. Mol. Biol. Rep. 47, 2529–2549. doi: 10.1007/s11033-020-05337-2, PMID: 32086721

[ref6] Booth T. H. Jovanovic T. Arnold R. J. (2017). Planting domains under climate change for *Eucalyptus pellita* and *Eucalyptus urograndis* in parts of China and South East Asia. Aust. For. 80, 1–9. doi: 10.1080/00049158.2016.1275101

[ref7] Brand A. Borovsky Y. Hill T. Rahman K. A. Bellalou A. Van Deynze A.. (2014). CaGLK2 regulates natural variation of chlorophyll content and fruit color in pepper fruit. Theor. Appl. Genet. 127, 2139–2148. doi: 10.1007/s00122-014-2367-y, PMID: 25096887

[ref8] Bravo-Garcia A. Yasumura Y. Langdale J. A. (2009). Specialization of the Golden2-like regulatory pathway during land plant evolution. New Phytol. 183, 133–141. doi: 10.1111/j.1469-8137.2009.02829.x, PMID: 19383092

[ref9] Browse J. Howe G. A. (2008). New weapons and a rapid response against insect attack. Plant Physiol. 146, 832–838. doi: 10.1104/pp.107.115683, PMID: 18316637 PMC2259070

[ref10] Chang D. Duda T. F. Jr. (2012). Extensive and continuous duplication facilitates rapid evolution and diversification of gene families. Mol. Biol. Evol. 29, 2019–2029. doi: 10.1093/molbev/mss068, PMID: 22337864

[ref11] Chen C. Chen H. Zhang Y. Thomas H. R. Frank M. H. He Y.. (2020). TBtools: an integrative toolkit developed for interactive analyses of big biological data. Mol. Plant 13, 1194–1202. doi: 10.1016/j.molp.2020.06.009, PMID: 32585190

[ref12] Chen M. Ji M. Wen B. Liu L. Li S. Chen X.. (2016). Golden 2-Like transcription factors of plants. Front. Plant Sci. 7, 1509. doi: 10.3389/fpls.2016.01509, PMID: 27757121 PMC5048441

[ref13] Chen H. Li J. Qiu B. Zhao Y. Liu Z. Yang J.. (2021). Long non-coding RNA and its regulatory network response to cold stress in *Eucalyptus urophylla* S.T.Blake. Forests 12, 836. doi: 10.3390/f12070836

[ref14] Deng X. Guo S. Sun L. Chen J. (2020). Identification of short-rotation *Eucalyptus* plantation at large scale using multi-satellite imageries and cloud computing platform. Remote Sens. 12, 2153. doi: 10.3390/rs12132153

[ref15] Deveaux Y. Peaucelle A. Roberts G. R. Coen E. Simon R. Mizukami Y.. (2003). The ethanol switch: a tool for tissue-specific gene induction during plant development. Plant J. 36, 918–930. doi: 10.1046/j.1365-313X.2003.01922.x, PMID: 14675455

[ref16] Du K. Liao T. Ren Y. Geng X. Kang X. (2020). Molecular mechanism of vegetative growth advantage in alotriploid *Populus*. Int. J. Mol. Sci. 21, 441. doi: 10.3390/ijms21020441, PMID: 32284503 PMC7014019

[ref17] Du K. Xia Y. Zhan D. Xu T. Lu T. Yang J.. (2022). Genome-wide identification of the *Eucalyptus urophylla* GATA gene family and its diverse roles in chlorophyll biosynthesis. Int. J. Mol. Sci. 23, 5251. doi: 10.3390/ijms23095251, PMID: 35563644 PMC9102942

[ref18] Fitter D. W. Martin D. J. Copley M. J. Scotland R. W. Langdale J. A. (2002). GLK gene pairs regulate chloroplast development in diverse plant species. Plant J. 31, 713–727. doi: 10.1046/j.1365-313X.2002.01390.x, PMID: 12220263

[ref19] Fujiwara T. Beachy R. N. (1994). Tissue-specific and temporal regulation of a β-conglycinin gene: roles of the RY repeat and other cis-acting elements. Plant Mol. Biol. 24, 261–272. doi: 10.1007/BF00020166, PMID: 8111031

[ref20] Gan P. Liu F. Li R. Wang S. Luo J. (2019). Chloroplasts-beyond energy capture and carbon fixation: tuning of photosynthesis in response to chilling stress. Int. J. Mol. Sci. 20, 5046. doi: 10.3390/ijms20205046, PMID: 31614592 PMC6834309

[ref21] Haas B. J. Salzberg S. L. Zhu W. Pertea M. Allen J. E. Orvis J.. (2008). Automated eukaryotic gene structure annotation using EVidenceModeler and the program to assemble spliced alignments. Genome Biol. 9, R7–R21. doi: 10.1186/gb-2008-9-1-r7, PMID: 18190707 PMC2395244

[ref22] Hii S. Y. Ha K. S. Ngui M. L. Ak Penguang S. Duju A. Teng X. Y.. (2017). Assessment of plantation-grown *Eucalyptus pellita* in Borneo, Malaysia for solid wood utilisation. Aust. For. 80, 26–33. doi: 10.1080/00049158.2016.1272526

[ref23] Jackson S. Chen Z. J. (2010). Genomic and expression plasticity of polyploidy. Curr. Opin. Plant Biol. 13, 153–159. doi: 10.1016/j.pbi.2009.11.004, PMID: 20031477 PMC2880571

[ref24] Kohl M. Wiese S. Warscheid B. (2011). Cytoscape: software for visualization and analysis of biological networks. Methods Mol. Biol. 696, 291–303. doi: 10.1007/978-1-60761-987-1_18 21063955

[ref25] Kumar S. Stecher G. Li M. Knyaz C. Tamura K. (2018). MEGA X: molecular evolutionary genetics analysis across computing platforms. Mol. Biol. Evol. 35, 1547–1549. doi: 10.1093/molbev/msy096, PMID: 29722887 PMC5967553

[ref26] Lai E. C. Burks C. Posakony J. W. (1998). The K box, a conserved 3′ UTR sequence motif, negatively regulates accumulation of enhancer of split complex transcripts. Development 125, 4077–4088. doi: 10.1242/dev.125.20.4077, PMID: 9735368

[ref27] Langdale J. A. Nelson T. (1991). Spatial regulation of photosynthetic development in C_4_ plants. Trends Genet. 7, 191–196. doi: 10.1016/0168-9525(91)90435-S, PMID: 1906211

[ref28] Li Y. Yang J. Song L. Qi Q. Du K. Han Q.. (2019). Study of variation in the growth, photosynthesis, and content of secondary metabolites in *Eucommia* triploids. Trees 33, 817–826. doi: 10.1007/s00468-019-01818-5

[ref29] Li X. Zhao L. Zhang H. Liu Q. Zhai H. Zhao N.. (2022). Genome-wide identification and characterization of CDPK family reveal their involvements in growth and development and abiotic stress in sweet potato and its two diploid relatives. Int. J. Mol. Sci. 23, 3088. doi: 10.3390/ijms23063088, PMID: 35328509 PMC8952862

[ref30] Liang Y. Xia J. Jiang Y. Bao Y. Chen H. Wang D.. (2022). Genome-wide identification and analysis of bZIP gene family and resistance of TaABI5 (TabZIP96) under freezing stress in wheat (*Triticum aestivum*). Int. J. Mol. Sci. 23, 2351. doi: 10.3390/ijms23042351, PMID: 35216467 PMC8874521

[ref31] Liao T. Cheng S. Zhu X. Min Y. Kang X. (2016). Effects of triploid status on growth, photosynthesis, and leaf area in *Populus*. Trees 30, 1137–1147. doi: 10.1007/s00468-016-1352-2

[ref32] Lim C. J. Lee H. Y. Kim W. B. Lee B.-S. Kim J. Ahmad R.. (2012). Screening of tissue-specific genes and promoters in tomato by comparing genome wide expression profiles of *Arabidopsis* orthologues. Mol. Cells 34, 53–59. doi: 10.1007/s10059-012-0068-4, PMID: 22699756 PMC3887779

[ref33] Lin Z. Li Q. Yin Q. Wang J. Zhang B. Gan S.. (2018). Identification of novel miRNAs and their target genes in *Eucalyptus grandis*. Tree Genet. Genomes 14, 1–9. doi: 10.1007/s11295-018-1273-x

[ref34] Lin Z. Long J. Yin Q. Wang B. Li H. Luo J.. (2019). Identification of novel lncRNAs in *Eucalyptus grandis*. Ind. Crop. Prod. 129, 309–317. doi: 10.1016/j.indcrop.2018.12.016

[ref35] Liu F. Xu Y. Han G. Zhou L. Ali A. Zhu S.. (2016). Molecular evolution and genetic variation of G2-Like transcription factor genes in maize. PLoS One 11:e0161763. doi: 10.1371/journal.pone.0161763, PMID: 27560803 PMC4999087

[ref36] Lupi A. C. D. Lira B. S. Gramegna G. Trench B. Alves F. R. R. Demarco D.. (2019). *Solanum lycopersicum* Golden 2-Like transcription factor affects fruit quality in a light- and auxin-dependent manner. PLoS One 14:e0212224. doi: 10.1371/journal.pone.0212224, PMID: 30753245 PMC6372215

[ref37] Menkens A. E. Schindler U. Cashmore A. R. (1995). The G-box: a ubiquitous regulatory DNA element in plants bound by the GBF family of bZIP proteins. Trends Biochem. Sci. 20, 506–510. doi: 10.1016/S0968-0004(00)89118-5, PMID: 8571452

[ref38] Mo L. Chen J. Lou X. Xu Q. Dong R. Tong Z.. (2020). Colchicine-induced polyploidy in *Rhododendron fortunei* Lindl. Plan. Theory 9, 424. doi: 10.3390/plants9040424, PMID: 32244298 PMC7238126

[ref39] Nakamura H. Muramatsu M. Hakata M. Ueno O. Nagamura Y. Hirochika H.. (2009). Ectopic overexpression of the transcription factor OsGLK1 induces chloroplast development in non-green rice cells. Plant Cell Physiol. 50, 1933–1949. doi: 10.1093/pcp/pcp138, PMID: 19808806 PMC2775961

[ref40] Nguyen C. V. Vrebalov J. T. Gapper N. E. Zheng Y. Zhong S. Fei Z.. (2014). Tomato Golden2-Like transcription factors reveal molecular gradients that function during fruit development and ripening. Plant Cell 26, 585–601. doi: 10.1105/tpc.113.118794, PMID: 24510723 PMC3967027

[ref41] Nie Y. M. Han F. X. Ma J. J. Chen X. Song Y. T. Niu S. H.. (2022). Genome-wide TCP transcription factors analysis provides insight into their new functions in seasonal and diurnal growth rhythm in *Pinus tabuliformis*. BMC Plant Biol. 22, 167. doi: 10.1186/s12870-022-03554-4, PMID: 35366809 PMC8976390

[ref42] Pappas Mde C. Pappas G. J. Jr. Grattapaglia D. (2015). Genome-wide discovery and validation of *Eucalyptus* small RNAs reveals variable patterns of conservation and diversity across species of Myrtaceae. BMC Genomics 16, 1113. doi: 10.1186/s12864-015-2322-6, PMID: 26714854 PMC4696225

[ref43] Pérez S. Renedo C. J. Ortiz A. Mañana M. Silió D. (2006). Energy evaluation of the *Eucalyptus globulus* and the *Eucalyptus nitens* in the north of Spain (Cantabria). Thermochim. Acta 451, 57–64. doi: 10.1016/j.tca.2006.08.009

[ref44] Powell A. L. T. Nguyen C. V. Hill T. Cheng K. L. Figueroa-Balderas R. Aktas H.. (2012). Uniform ripening encodes a Golden 2-Like transcription factor regulating tomato fruit chloroplast development. Science 336, 1711–1715. doi: 10.1126/science.1222218, PMID: 22745430

[ref45] Qin M. Zhang B. Gu G. Yuan J. Yang X. Yang J.. (2021). Genome-wide analysis of the G2-like transcription factor genes and their expression in different senescence stages of tobacco (*Nicotiana tabacum* L.). Front. Genet. 12:626352. doi: 10.3389/fgene.2021.626352, PMID: 34135936 PMC8202009

[ref46] Riechmann J. L. Heard J. Martin G. Reuber L. Jiang C.-Z. Keddie J.. (2000). *Arabidopsis* transcription factors: genome-wide comparative analysis among eukaryotes. Science 290, 2105–2110. doi: 10.1126/science.290.5499.2105 11118137

[ref47] Rossini L. Cribb L. Martin D. J. Langdale J. A. (2001). The maize Golden2 gene defines a novel class of transcriptional regulators in plants. Plant Cell 13, 1231–1244. doi: 10.1105/tpc.13.5.1231, PMID: 11340194 PMC135554

[ref48] Roulin A. Auer P. L. Libault M. Schlueter J. Farmer A. May G.. (2013). The fate of duplicated genes in a polyploid plant genome. Plant J. 73, 143–153. doi: 10.1111/tpj.12026, PMID: 22974547

[ref49] Song H. Wang P. Lin J.-Y. Zhao C. Bi Y. Wang X. (2016). Genome-wide identification and characterization of WRKY gene family in peanut. Front. Plant Sci. 7:534. doi: 10.3389/fpls.2016.00534, PMID: 27200012 PMC4845656

[ref50] Thompson J. D. Gibson T. J. Plewniak F. Jeanmougin F. Higgins D. G. (1997). The CLUSTAL_X windows interface: flexible strategies for multiple sequence alignment aided by quality analysis tools. Nucleic Acids Res. 25, 4876–4882. doi: 10.1093/nar/25.24.4876, PMID: 9396791 PMC147148

[ref51] Unnikrishnan B. V. Shankaranarayana G. D. (2020). Insights into microRNAs and their targets associated with lignin composition in *Eucalyptus camaldulensis*. Plant Gene 24:100248. doi: 10.1016/j.plgene.2020.100248

[ref52] Valli H. Prasanna D. Rajput V. S. Sridhar W. Sakuntala N. N. V. Harshavardhan P.. (2022). Cis elements: added boost to the directed evolution of plant genes. J. Pure Appl. Microbiol. 16, 663–668. doi: 10.22207/jpam.16.1.68

[ref53] Vilasboa J. Da Costa C. T. Fett-Neto A. G. (2019). Rooting of eucalypt cuttings as a problem-solving oriented model in plant biology. Prog. Biophys. Mol. Biol. 146, 85–97. doi: 10.1016/j.pbiomolbio.2018.12.007, PMID: 30557533

[ref54] Wang P. Fouracre J. Kelly S. Karki S. Gowik U. Aubry S.. (2013). Evolution of Golden 2-Like gene function in C_3_ and C_4_ plants. Planta 237, 481–495. doi: 10.1007/s00425-012-1754-3, PMID: 22968911 PMC3555242

[ref55] Wang Y. Liu G. J. Yan X. F. Wei Z. G. Xu Z. R. (2011). MeJA-inducible expression of the heterologous JAZ2 promoter from Arabidopsis in *Populus trichocarpa* protoplasts. J. Plant Dis. Prot. 118, 69–74. doi: 10.1007/BF03356384

[ref56] Wang Y. Tang H. Debarry J. D. Tan X. Li J. Wang X.. (2012). MCScanX: a toolkit for detection and evolutionary analysis of gene synteny and collinearity. Nucleic Acids Res. 40:e49. doi: 10.1093/nar/gkr1293, PMID: 22217600 PMC3326336

[ref57] Wang Z. Y. Zhao S. Liu J. F. Zhao H. Y. Sun X. Y. Wu T. R.. (2022). Genome-wide identification of Tomato Golden 2-Like transcription factors and abiotic stress related members screening. BMC Plant Biol. 22, 82. doi: 10.1186/s12870-022-03460-9, PMID: 35196981 PMC8864820

[ref58] Waters M. T. Moylan E. C. Langdale J. A. (2008). GLK transcription factors regulate chloroplast development in a cell-autonomous manner. Plant J. 56, 432–444. doi: 10.1111/j.1365-313X.2008.03616.x, PMID: 18643989

[ref59] Waters M. T. Wang P. Korkaric M. Capper R. G. Saunders N. J. Langdale J. A. (2009). GLK transcription factors coordinate expression of the photosynthetic apparatus in *Arabidopsis*. Plant Cell 21, 1109–1128. doi: 10.1105/tpc.108.065250, PMID: 19376934 PMC2685620

[ref60] Wu S. Wu M. Dong Q. Jiang H. Cai R. Xiang Y. (2016). Genome-wide identification, classification and expression analysis of the PHD-finger protein family in *Populus trichocarpa*. Gene 575, 75–89. doi: 10.1016/j.gene.2015.08.042, PMID: 26314912

[ref61] Xiao Y. You S. Kong W. Tang Q. Bai W. Cai Y.. (2019). A GARP transcription factor anther dehiscence defected 1 (*OsADD1*) regulates rice anther dehiscence. Plant Mol. Biol. 101, 403–414. doi: 10.1007/s11103-019-00911-0, PMID: 31420780

[ref62] Yang X. Guo T. Li J. Chen Z. Guo B. An X. (2021). Genome-wide analysis of the MYB-related transcription factor family and associated responses to abiotic stressors in *Populus*. Int. J. Biol. Macromol. 191, 359–376. doi: 10.1016/j.ijbiomac.2021.09.042, PMID: 34534587

[ref63] Yang J. Wang J. Liu Z. Xiong T. Lan J. Han Q.. (2018). Megaspore chromosome doubling in *Eucalyptus urophylla* S.T. Blake induced by colchicine treatment to produce triploids. Forests 9, 728. doi: 10.3390/f9110728

[ref64] Zhang J. (2003). Evolution by gene duplication: an update. Trends Ecol. Evol. 18, 292–298. doi: 10.1016/S0169-5347(03)00033-8

[ref65] Zhang J. Liu Y. Xia E. H. Yao Q. Y. Liu X. D. Gao L. Z. (2015). Autotetraploid rice methylome analysis reveals methylation variation of transposable elements and their effects on gene expression. Proc. Natl. Acad. Sci. U. S. A. 112, E7022–E7029. doi: 10.1073/pnas.1515170112, PMID: 26621743 PMC4687577

[ref66] Zubo Y. O. Blakley I. C. Franco-Zorrilla J. M. Yamburenko M. V. Solano R. Kieber J. J.. (2018). Coordination of chloroplast development through the action of the GNC and GLK transcription factor families. Plant Physiol. 178, 130–147. doi: 10.1104/pp.18.00414, PMID: 30002259 PMC6130010

