# Supplementary material for: Genome-wide characterization and analysis of Golden 2-Like transcription factors related to leaf chlorophyll synthesis in diploid and triploid Eucalyptus urophylla
Source: Front Plant Sci. 2022 Jul 28;13:952877. doi: 10.3389/fpls.2022.952877 (PMC9366356; doi:10.3389/fpls.2022.952877)
Supplement: Supplementary file 1 [file Data_Sheet_1.docx]

Supplementary Figures


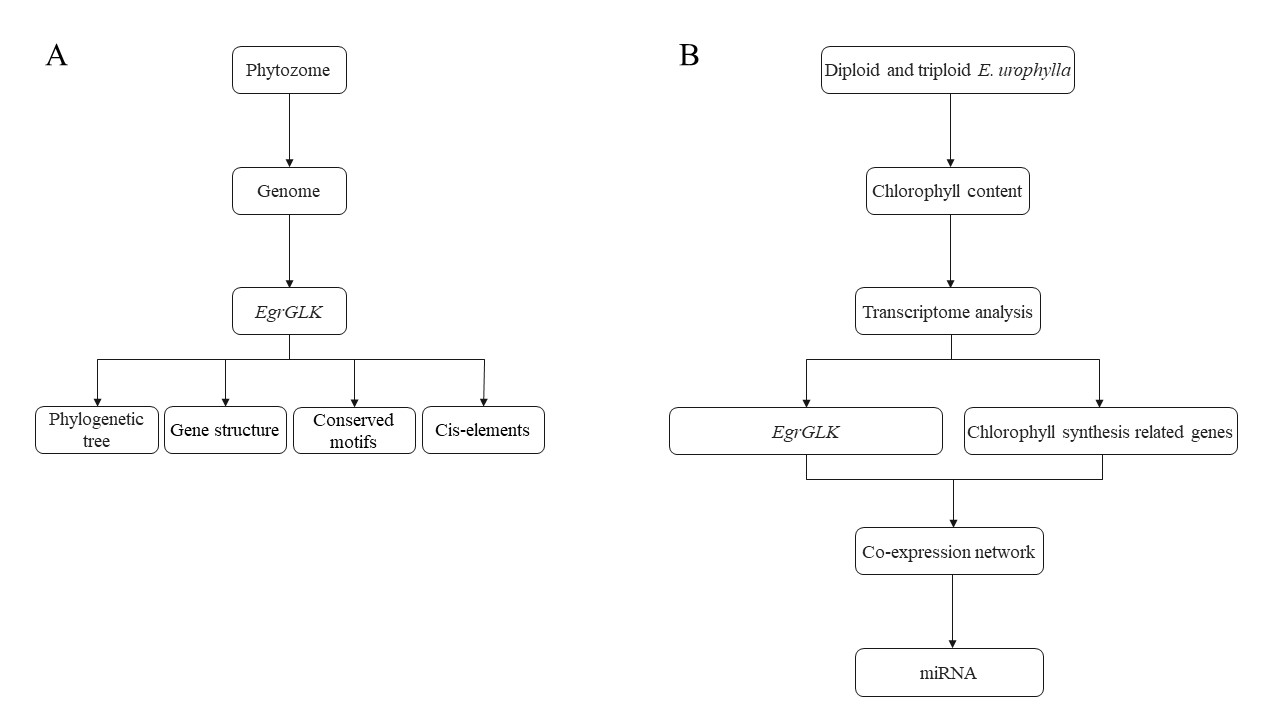


FIGURE S1 The framework figure of the analysis.

(**A**) Identification and bioinformatics analysis of *EgrGLK* gene. (**B**) Chlorophyll content and transcriptome analysis of diploid and triploid *E. urophylla.*


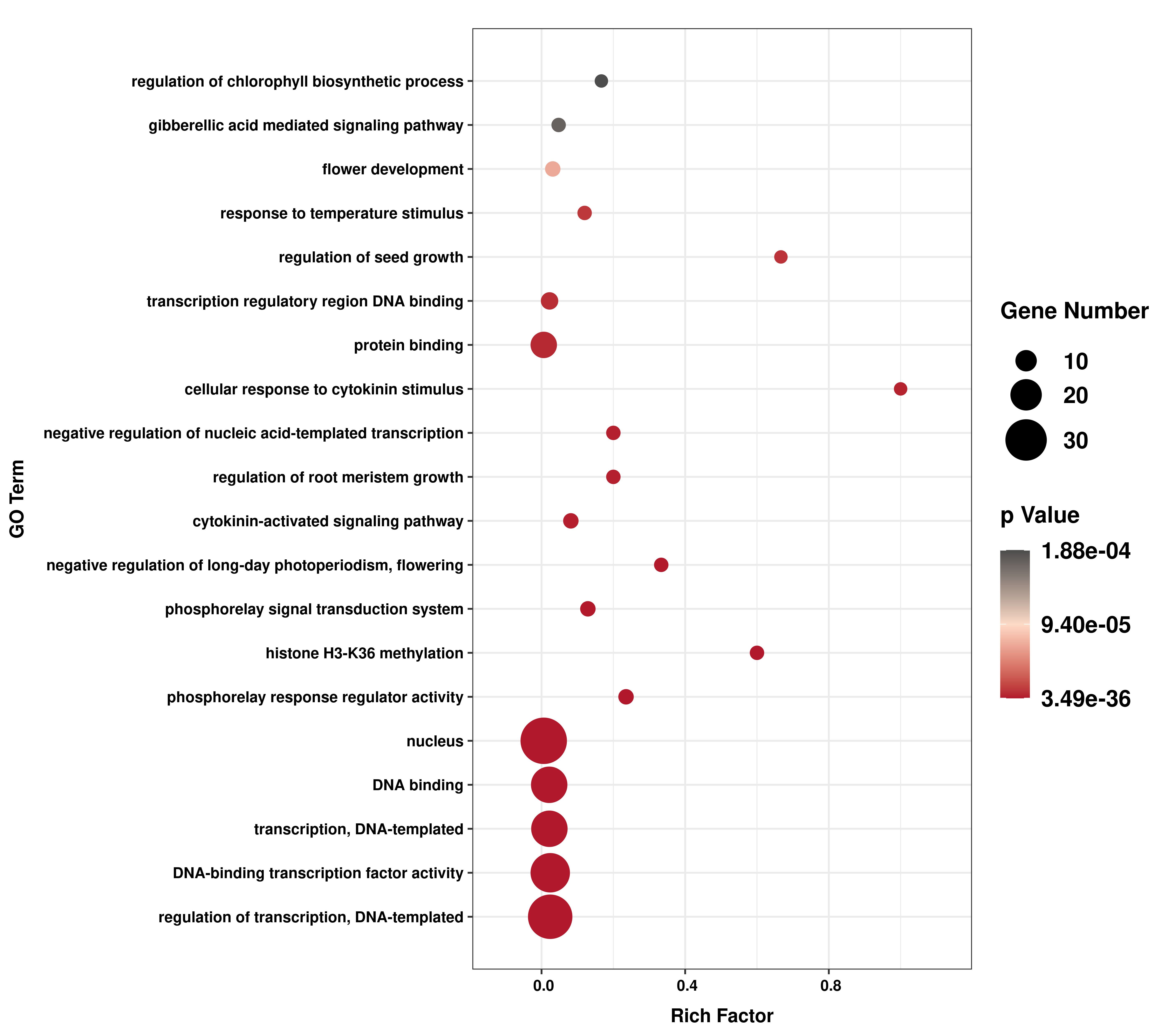


FIGURE S2 GO annotation of *EgrGLK* genes in *E. urophylla* leaves*.*


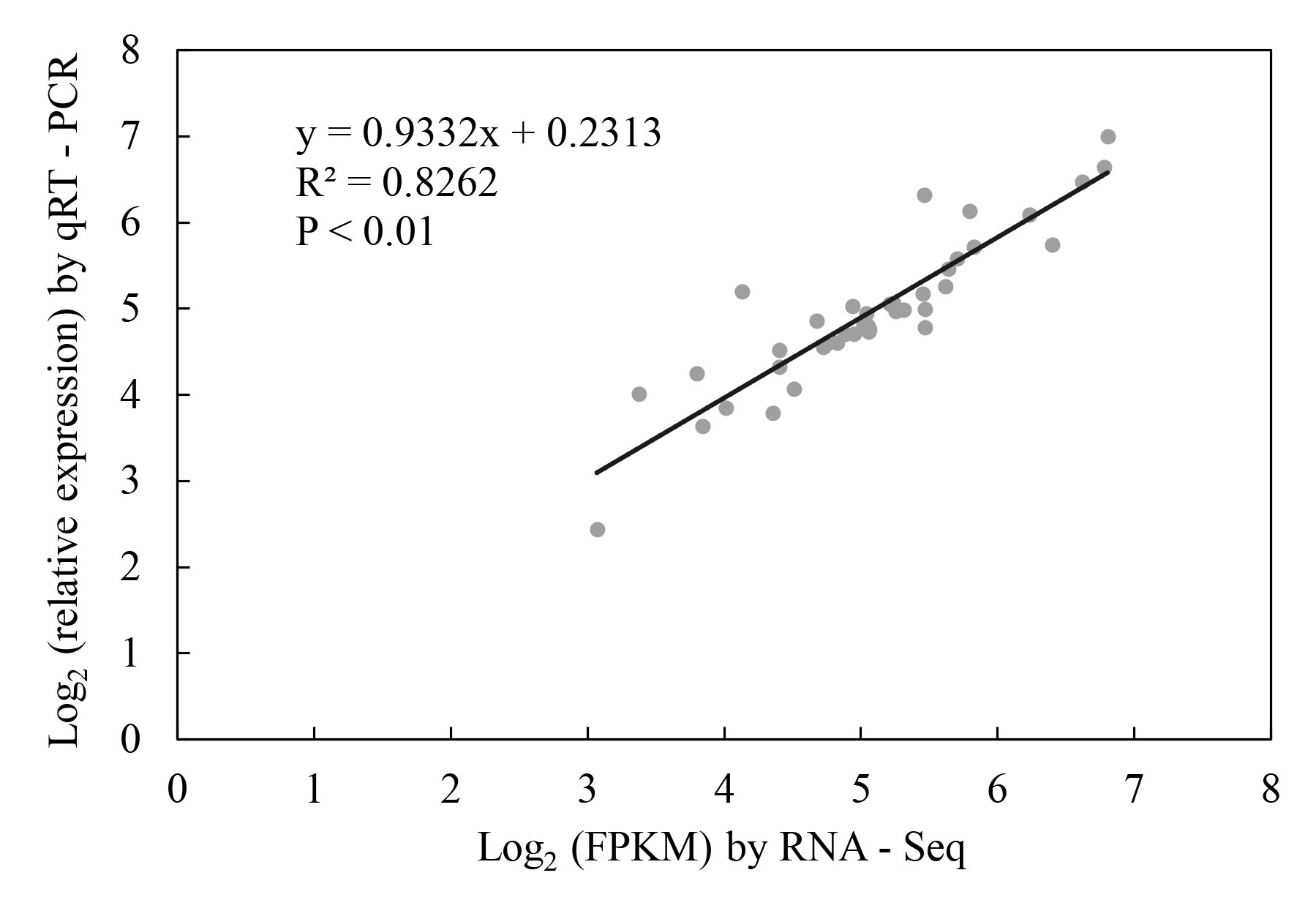


FIGURE S3 Correlation of expression between RNA-seq (X-axis) and qPCR (Y-axis).
